# Is sCD163 a Clinical Significant Prognostic Value in Cancers? A Systematic Review and Meta-Analysis

**DOI:** 10.3389/fonc.2020.585297

**Published:** 2020-11-10

**Authors:** Shushu Qian, Hong Zhang, Huibo Dai, Bangyun Ma, Fang Tian, PengJun Jiang, Haoran Gao, Xiaocao Sha, Xuemei Sun

**Affiliations:** ^1^ Department of Hematology, Affiliated Hospital of Nanjing University of Chinese Medicine, Nanjing, China; ^2^ Department of Center Laboratory, Affiliated Hospital of Nanjing University of Chinese Medicine, Nanjing, China

**Keywords:** soluble CD163, tumor associated macrophages, meta-analysis, prognostic value, tumor microenvironment

## Abstract

**Background:**

Tumor associated macrophages (TAMs), a kind of inflammatory cells in the tumor microenvironment, are crucial for the occurrence and development of various tumors which increased the expression of CD163. Nevertheless, not much has been established regarding soluble CD163 and its connection to tumor diagnosis. In this case, a meta-analysis was conducted to determine the tumor diagnostic importance of serum sCD163.

**Methods:**

In order to assess the correlation between sCD163 and the overall survival (OS) or progression-free survival (PFS) among tumor patients, a systematic perusal of literature published until June 2020 was conducted. Relevant data were primarily obtained from papers that have the following qualifications: 1) a confidence interval (CI) of 95%; 2) a report of the hazard ratios; and, 3) pooled by means of the Mantel–Haenszel random-effect representation.

**Results:**

For the final meta-analysis, eight papers comprised of 1,236 cases were involved. Through pooled investigation, it was determined that a correlation exists between elevated serum sCD163 and worse OS (HR = 2.24, 95% CI: 1.50–3.35, *P* < 0.001) and PFS (HR = 3.90, 95% CI: 2.33–6.52, *P* < 0.001) among tumor cases. Subgroup analysis stratified by medium age at diagnosis demonstrated that patients over 60 years old with high sCD163 had worse OS (HR 2.28, 95% CI: 1.58–3.29, P < 0.001) than under 60 (HR 1.43, 95% CI: 1.15–1.77, *P* = 0.001). Subgroup analysis revealed that analysis method and medium age at diagnosis were the potential source of heterogeneity.

**Conclusions:**

Overall, diagnosis of tumor cases can be adversely determined through substantial sCD163 levels. Consequently, it is encouraged that extensive researches regarding the rates of cancer survival be accomplished.

## Introduction

Incidences of carcinoma increase globally year by year and it has been considered a crucial public health concern threatening the safety of human life. Along with scientific advancement, cancer detection, and treatment methods have been greatly improved, leading to a considerable prolonging of patient survival time. However, the prognosis of patients with advanced cancer is still very poor, and the mortality rate is very high (1). Of particular interest to this study, in recent years, researchers have made significant efforts in finding markers related to tumor prognosis to help clinicians make accurate judgments on conditions of cancer patients. Furthermore, this has led to assisting doctors in clinical decision-making, so as to improve the survival time of patients (2).

Tumor-associated macrophages (TAMs) are a class of inflammatory cells in the tumor microenvironment which participate in the growth, angiogenesis, and metastasis of tumor cells (3). It has been reported that macrophage infiltration is closely correlated to the prognosis of tumors (4). Macrophages display two kinds of polarization states. The alternatively activated type 2 macrophages (M2) with an anti-inflammatory effect can produce immunosuppressive and tumor-promoting factors, such as IL-6, IL-10, vascular endothelial growth factor, and several metalloproteinases (5–7). CD163 is a scavenger receptor of monocyte macrophages, which is related to reduced inflammation (8), and TAMs increased the expression of CD163. Soluble CD163 (sCD163) is produced by proteolysis of membrane proteins and then released into serum or other body fluids in a soluble form (5).

It is worth noting that the current published studies show that in the serum of tumor patients serum sCD163 levels are elevated, and it can be used to estimate the total-body M2 macrophage load (8). Further, some studies show that increased levels of serum sCD163 are linked to poor prognosis (9–12). However, other studies contradict this by showing that the level of serum sCD163 has no relationship with the prognosis of tumors (13). The prognostic value of serum sCD163 level in tumors remains controversial. It is generally acknowledged that meta-analysis is a powerful static tool for generating the best estimate by overcoming the limitation of different sample sizes from individual studies. On this basis, this paper intends to conduct a meta-analysis of serum sCD163 by expanding the number of studies and samples, to explore its role in tumor prognosis.

## Materials and Methods

### Search Strategy

Articles published before June 8, 2020, were extracted from PubMed, the Web of Science, Embase, CBM, Scopus, and Cochrane. We used the MeSH terms and keywords of “Neoplasms or Neoplasia or Tumor or Cancer or Malignancy or Neoplasms Malignancy” and “CD163 or sCD163 or soluble CD163 or soluble scavenger receptor” in our searches. Likewise, in order to acquire further pertinent researches as vital for analysis of data, a list of possible English-published crux studies was also perused (see **Supplementary Table 1** for the search strategies).

### Section Criteria

Qualifications were set out for selecting the researches, identified as follows: the inclusion conditions to undertake the meta-analysis comprise: i) statistics on the relationship between serum sCD163 and overall survival (OS) or progression-free survival (PFS) in patients with cancer; ii) sCD163 was measured by serum based methods; iii) HR with 95% confidence interval (CI) was determined as reported by the researches themselves, or as computed through adequate data. Exclusion criteria were as follows: i) duplicated publications or; ii) studies were not written in English or; iii) letters, case reports, and reviews or; iv) sample size <20.

### Data Extraction and Quality Assessment

Two autonomous authors—SQ and HZ—obtained and assessed the initially chosen papers. Those papers that required further assessment even after perusing the respective titles and abstracts were then classified after a complete scrutiny of the text. Should a discrepancy arise between the two authors, they would convene and reach an agreement with the presence of a third author (HD). For each study, the following items was recorded: first author, year of publication, study region, study design, inclusion period, cancer type, total number of cases and gender, cut-off value, follow-ups, survival outcome, and HRs with 95% CIs. The Newcastle-Ottawa Scale (NOS) was used to assess each of the included studies quality by two independent authors (SQ and HZ). The NOS consists of three parts: selection (0–4 points), comparability (0–2 points), and outcome assessment (0–3 points). NOS scores of 6 were assigned as high-quality studies.

### Statistical Analysis

Pooled HRs with 95% CIs were calculated to investigate the value of serum sCD163 on the survival of patients with cancer. Pooled ORs with 95% CIs were used to evaluate the association between the level of serum sCD163 and clinicopathological features. The heterogeneity of the pooled results was explored by means of the Q test and I^2^ statistics. The fixed pooling model was selected when I^2^ < 50% and *P* > 0.10. Otherwise, the random pooling model was used. Subgroup analysis was further conducted in order to determine the possible heterogeneity causes. To evaluate if a certain research could affect the overall outcomes’ stability, sensitivity analysis was undertaken. Meanwhile, the Egger’s test was applied to measure biases in publication, if any. To be deemed statistically significant, a value of p that was less than 0.05 was necessary.

## Results

### Study Selection and Features

**Figure 1:**
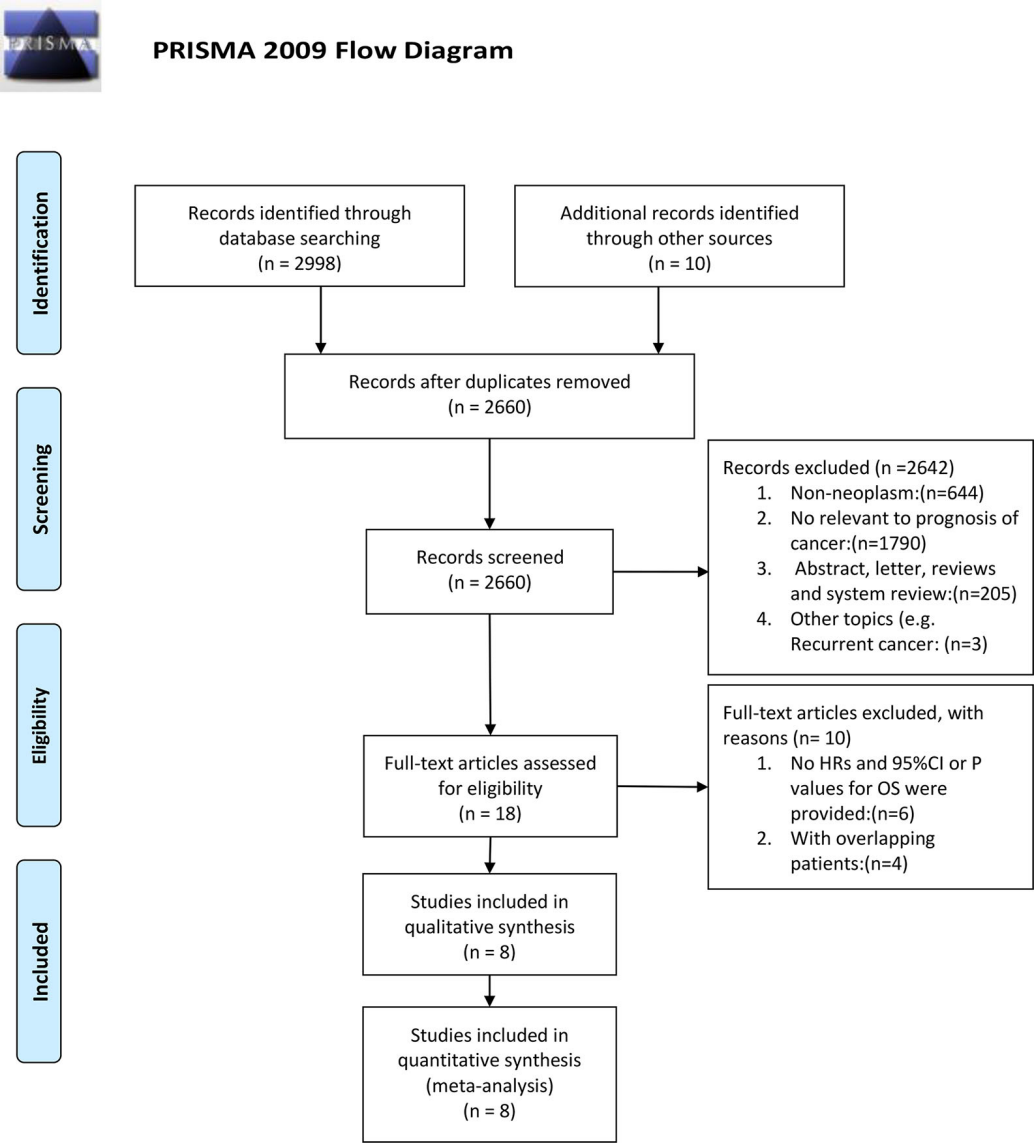
PRISMA 2009 flow diagram.

Basing from the aforementioned selection standards, eight papers were qualified for meta-analysis. In total, 1,236 patients with seven types of cancer, including multiple myeloma (MM) (12), classic Hodgkin lymphoma (cHL) (14), gastric cancer (GC) (15), melanoma (16), hepatocellular carcinoma (HCC) (9, 17), epithelial ovarian cancer (10), and B-cell lymphocytic leukemia (11), were analyzed. Four articles chose overall survival (OS) as the only survival outcome (12, 15–17), one article used both OS and progression-free survival (PFS) (9), one article used both OS and disease-free survival (DFS) (10), and one article used both OS and failure-free survival (FFS) (14). One article chose progression-free survival (PFS) as the only survival outcome (11). Among the eight selected studies, three were from Denmark, one from Germany, one each from USA, north America, China, Australia, and Korea. The concentration of sCD163 were measured using an enzyme-linked immunosorbent assay or multiplex (Luminex) bead array immunoassay in peripheral blood. The NOS scores of the included studies ranged from 5 to 9, only one study under 7, suggesting a relatively high quality (**Supplementary Figure 8**). The screening process is summarized as a PRISMA flow diagram (18) (**Figure 1**). More details are shown in **Table 1**.

**Table 1:** Main characteristics of all the studies included in the meta-analysis.

No.	Authors	Year	Study region	Study design	Inclusion period	Disease	No. (M/F)	Age (years)	Cut-off(µg/ml)	Medium and range(µg/ml)	Follow-up(months)	Outcome	HR	NOS
1	Morten NA (12)	2014	Denmark	Retrospective	1991–2004	Multiple myeloma	104 (56/48)	69	1.8	2.09 (1.52–2.84)	36 (1–159)	OS	R (U/M)	8
2	DONG BD (15)	2017	China	Prospective	2012–2013	Gastric cancer	143 (98/45)	64 (35–89)	0.6645	0.628 (0.291~1.76)	40	OS	R (U/M)	9
3	Trine OJ (16)	2009	Denmark	Prospective	1997–2000	Melanoma	227 (113/114)	55 (25–86)	NR	NR	60	OS	R (U/M)	9
4	Konstantin K (9)	2015	Australia	Prospective	2008–2010	hepatocellular carcinoma	109 (96/13)	63 (55–71)	8	5.6 (3.5–8.0)	8.1	OS/PFS	N (U)	8
5	Jae HN (10)	2013	Korea	Prospective	2005–2009	Epithelial ovarian cancer	55	53.5 (41–66)	3.43	1.71 (1.05–2.86)	NR	OS	R (M)	8
6	Oliver W (17)	2013	Germany	Prospective	2009–2011	Hepatocellular carcinoma	267 (220/47)	64.4	3.9	NR	282	OS	R (U/M)	9
7	Kanakry JA (14)	2016	North America	Prospective	1994–2006	Classical Hodgkin lymphoma	301	NR	NR	NR	NR	OS	R (U)	5
8	Nederby L (11)	2014	Denmark	Prospective	2014	B-cell lymphocytic leukemia	30	NR	2.45	2.085 (0.77–9.01)	24	PFS	R (U)	7

OS, overall survival; PFS, progression-free survival; PFS, progression-free survival; HR, hazard ratio, obtained by reporting in text (R). “M” means the HR come from multivariate analysis, “U” means the HR comes from univariate analysis; NR, not reported; NOS, Newcastle-Ottawa Quality Assessment Scale.

### Meta-Analysis of the Association Between the Levels of sCD163 and Survival

**Figure 2:**
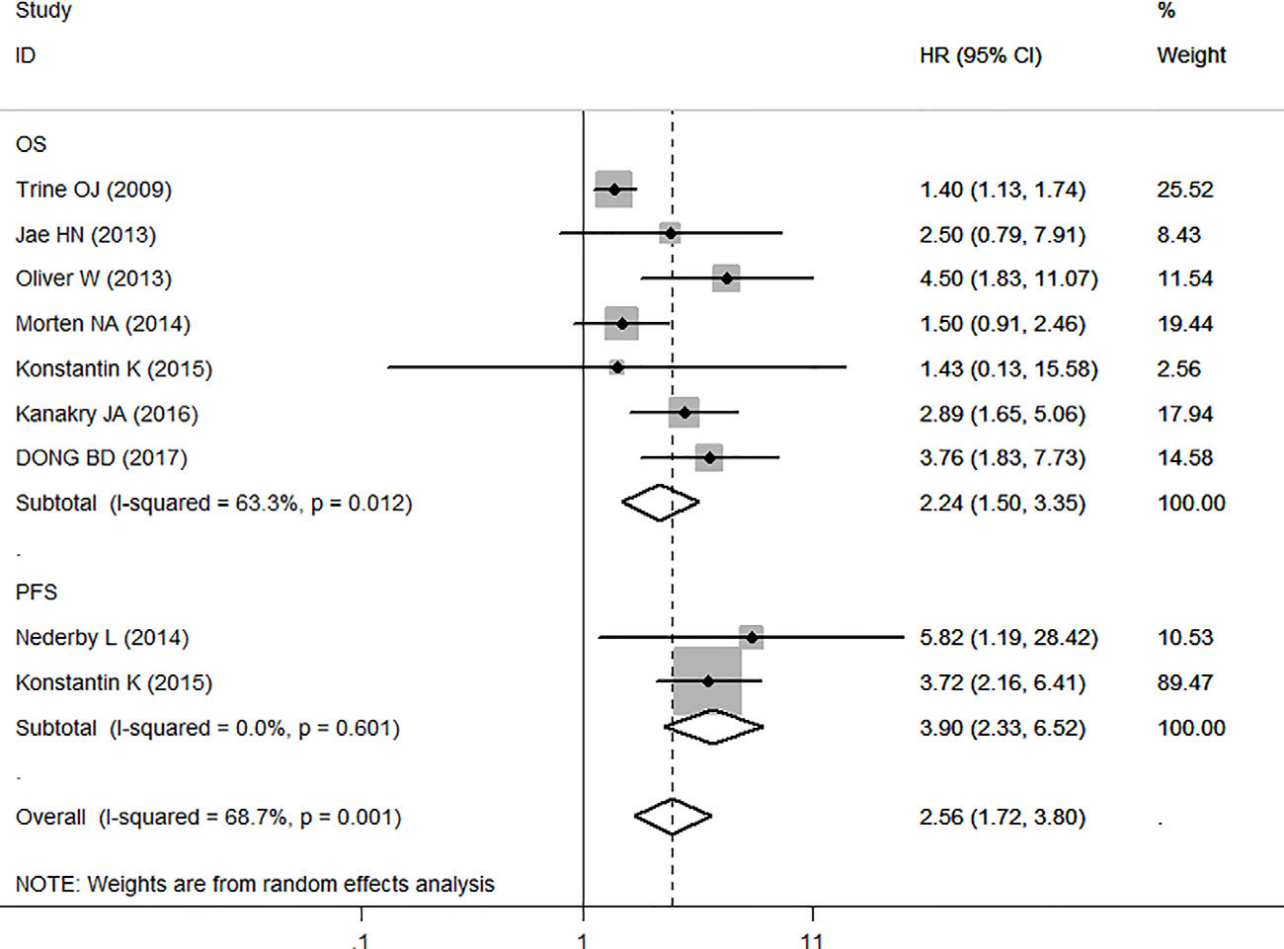
Meta-analysis of the association between sCD163 and overall survival (OS)/progression-free survival (PFS) of cancer. Results are presented as individual and pooled HR, and 95% CI.

Seven studies with 1,206 individuals were included in the analysis of the association between sCD163 and OS. The random-effects model was used in this meta-analysis because of the heterogeneity test (I^2^ = 63.3%, *P* < 0.001). It showed that elevated serum sCD163 levels were significantly associated with poor OS in cancer patients (HR = 2.24, 95% CI: 1.50–3.35, *P* < 0.001) (**Figure 2**).

To explain the heterogeneity in OS, subgroup analysis was performed by examining sCD163 levels in sample size (sample size ≥ 200 or sample size ≥ 200), analysis method (multivariate analysis or univariate analysis), cancer type (solid tumor or hematological tumor), and medium age at diagnosis (age ≥ 60 or age ≥ 60) as shown in **Table 2**. The result of each subgroup was not significantly changed. Subgroup analysis stratified by medium age at diagnosis demonstrated that patients over 60 years old with high sCD163 had worse OS (HR 2.28, 95% CI: 1.58–3.29, *P* < 0.001) than under 60 (HR 1.43, 95% CI: 1.15–1.77, *P* = 0.001). Subgroup analysis revealed that analysis method and medium age at diagnosis were the potential source of heterogeneity (**Supplementary Figures 1–5**).

**Table 2:** Summary of the meta-analysis results.

Analysis	N	References	Random-effects model		Fixed-effects model		Heterogeneity	
			HR (95% CI)	P	HR (95% CI)	P	*I^2^*	*Ph*
OS	7	(9, 10, 12, 14–17)	2.24 (1.50, 3.35)	0	1.70 (1.43, 2.03)	0	63.3%	0.012
Subgroup 1: sample size ≥ 200	2	(9, 15)	1.92 (0.95, 3.88)	0.07	2.30 (1.62, 3.26)	0	82.1%	0.018
Sample size ≥ 200	5	(10, 12, 14, 16, 17)	2.56 (1.51, 4.32)	0	1.54 (1.26, 1.89)	0	42.5%	0.138
Subgroup 2: univariate analysis	3	(9, 14, 16)	3.11 (2.01, 4.80)	0	3.11 (2.01, 4.80)	0	0.0%	0.692
Multivariate analysis	4	(10, 12, 15, 17)	1.83 (1.19, 2.82)	0.006	1.52 (1.25, 1.84)	0	56.2%	0.077
Subgroup 3: solid tumor	5	(10, 14, 15, 16, 17)	2.43 (1.31, 4.68)	0.005	1.63 (1.33, 1.99)	0	67.9%	0.014
Hematological tumor	2	(9, 12)	2.05 (1.08, 3.90)	0.028	2.00 (1.38, 2.90)	0	66.2%	0.085
Subgroup 4: medium age at diagnosis ≥ 60	4	(12, 14, 16, 17)	2.61 (1.36, 5.01)	0.004	2.28 (1.58, 3.29)	0	56.7%	0.074
Medium age at diagnosis ≥60	2	(10, 15)	1.43 (1.15, 1.77)	0.001	1.43 (1.15, 1.77)	0.001	0.0%	0.332
PFS	2	(11, 16)	3.90 (2.33, 6.52)	0	3.90 (2.33, 6.52)	0	0.0%	0.601

N, number of studies; HR, hazard ratio; 95% CI, 95% confidence interval; Ph, P values of Q test; OS, overall survival; PFS, progression free survival.

We also made this meta-analysis to find out the role of sCD163 from the eligible 2 studies in PFS of 139 tumor patients (**Figure 2**). A fixed-effect model was then used with no significant heterogeneity (I^2^ = 0.0%, *P* = 0.601). The analyses showed that high levels of sCD163 was related to worse PFS (HR = 3.90, 95% CI: 2.33,6.52, *P* < 0.001).

### Publication Bias and Sensitivity Analysis

**Figure 3:**
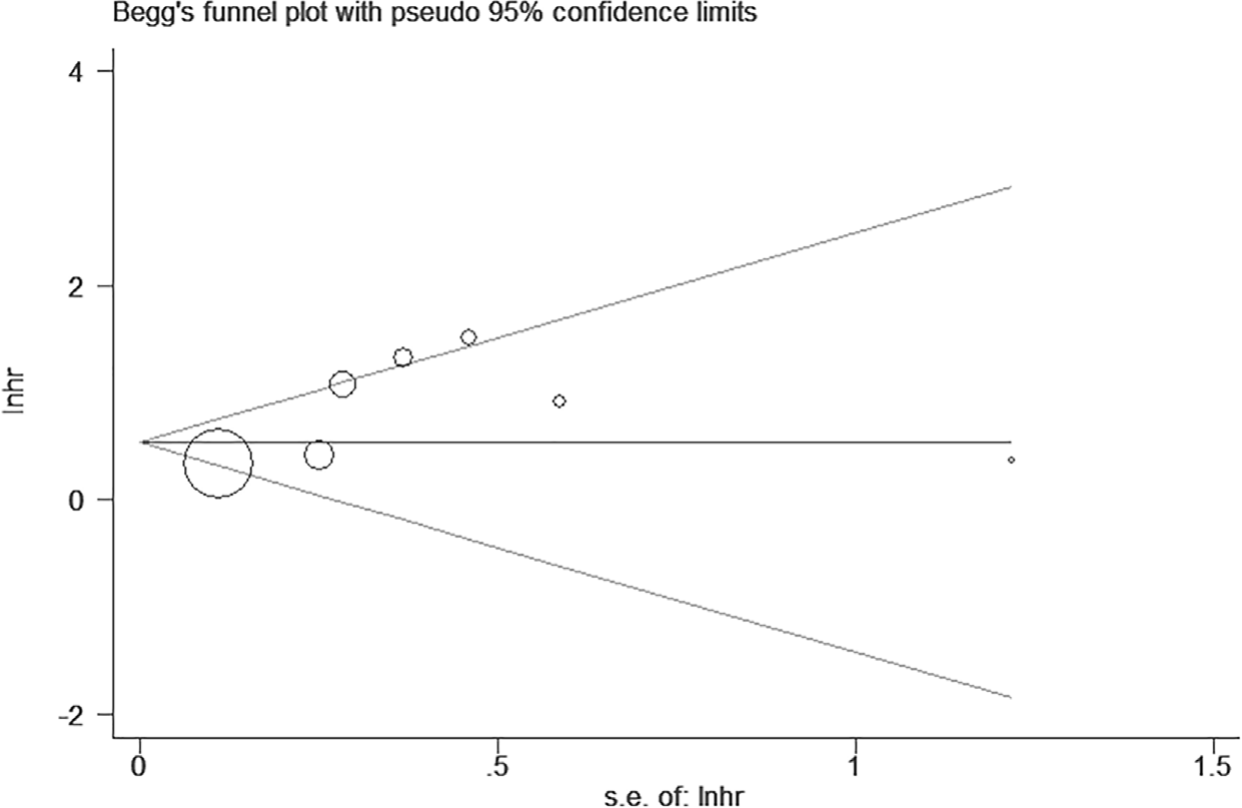
Begg’s test for publication bias of results of survival.

**Figure 4:**
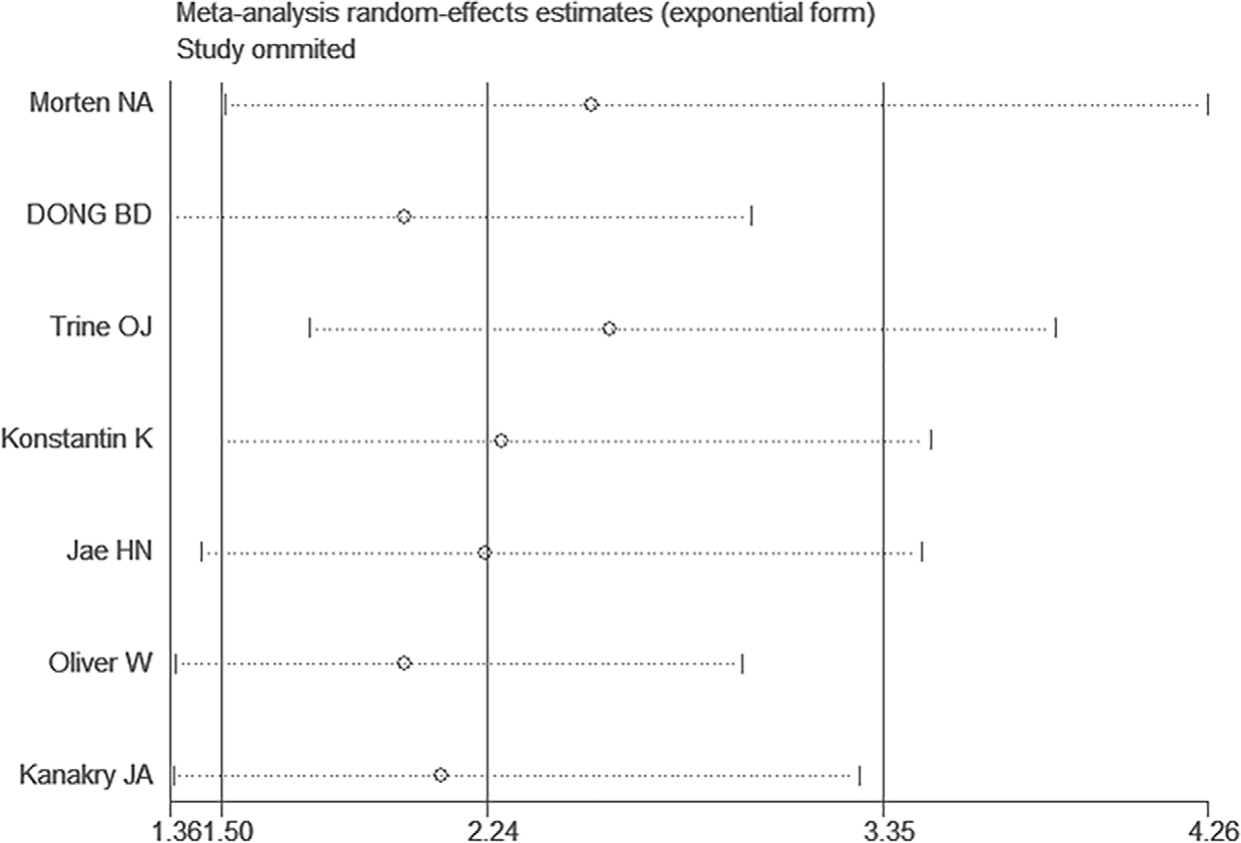
Sensitivity analysis for studies about overall survival (OS) by omitting each study sequential.

In order to probe any sort of bias in terms of publication, the Begg’s test was performed. Basing from **Figure 4**, the resulting Begg’s symmetrical plot accounting for the eight qualified articles on survival did not manifest any significant bias on publication, with a P-value equal to 0.548 (**Figure 3**) (**Supplementary Figures 6**).

Afterward, to evaluate the degree of stability of the initial OS outcomes, it was necessary to undertake sensitivity analysis. In this case, the meta-analysis was validated to have exhibited robustness, since the outcomes had not substantially changed even after any article was removed (**Figure 4**) (**Supplementary Figure 7**).

## Discussion

The contemporary innovations in tumor treatment and detection have rendered considerable focus regarding the determination of possible prognostic biomarkers among patients in order to enhance the survival and efficacy rates, particularly by utilizing relevant information (19, 20). Regarding most tumor types, cancer patients have still generally exhibited comparably low rates of survival. Hence, the current paper intends to demonstrate a meta-analysis.

The microenvironment of a tumor remains a vital basis for its behavior among several cancerous ailments; correspondingly, TAMs or tumor-associated macrophages are major cellular components of tumors as they play a pivotal function in carcinoma development. Medically speaking, this interaction has been depicted *via* a determined effect on a tumor’s progression by the densities of TAMs (21, 22). Accordingly, TAMs can signify potential medicinal objectives for cancers, as macrophage-related indicators have posed themselves as likely prognosticators involving cancer existence and development. CD163, a monocyte-macrophage scavenger receptor can be regarded as a macrophage-activation marker. Meanwhile, its soluble form, sCD163, is produced through proteolytic cleavage involving a membrane protein, before shedding as a soluble type onto a tissue fluid or serum (23). Remarkably, this specific form is greatly associated with various ailments, such as ovarian cancer, liver cancer, multiple myeloma, and leukemia. Therefore, sCD163 may be identified as a negative prognostic factor for these cancers.

The level of sCD163 will be affected by the up-regulation of CD163 surface expression, the increase of exfoliation and the change of its clearance rate (24). The expression and secretion of CD163 on the cell surface are regulated by the stimulation of different cytokines. It is markedly induced by anti-inflammatory mediators, such as glucocorticoids and interleukin-10, and leads to increased serum levels of sCD163, whereas it is inhibited by proinflammatory mediators, such as interferon-γ and TNF (25, 26). It is generally considered that a disintegrin and metalloproteinase 17 (ADAM17)/TNF-α–cleaving enzyme (TACE) is the responsible enzyme for cleavage of CD163 (27–29). Furthermore, several inflammatory signals have manifested an inducement involving *in vitro* sCD163 ectodomain shedding. Shedding can be induced by Toll like receptor (TLR) activation by LPS (lipopolysaccharide) or PMA (phorbol 12-myristate 13-acetate), Fcγ receptor-crosslinking mediated by ADAM17/TACE, oxidative stress, and thrombin (26). Presently, there is limited knowledge about sCD163 and the factors influencing its shedding. The level of sCD163 regulated by the tumor microenvironment will be better explained with deeper further research.

A total of eight qualified articles that had 1,236 cases involving cancer comprised the meta-analysis, which employed a fixed-effects or random model corresponding to an examination on heterogeneity. The combined HR revealed that a higher level of sCD163 was significantly correlated with shorter OS and PFS of patients with different kinds of cancer. The association of sCD163 with OS was robust in both solid tumors and hematological tumors. A subgroup analysis of OS indicated no significant change in any subgroup. On account of the limited number of studies and sample sizes for each cancer site, these conclusions require additional prospective articles for verification.

Two studies of the eight articles included in this meta-analysis pointed out that sCD163 levels decreased after the treatment, while the other six did not mention the relationship between sCD163 levels and treatment. According to the results of these two studies, the sCD163 level of multiple myeloma patients in the study decreased 3 months after the HDT treatment (high-dose melphalan with autologous stem cell support); however, the sCD163 level after HDT treatment was not related to the efficacy of HDT (12). The other study indicated that after 4 weeks of radiofrequency ablation, the sCD163 level of hepatocellular carcinoma decreased and the sCD163 level of patients with advanced disease was higher than that of patients with stable condition and regression (9). It is roughly concluded that the sCD163 levels of cancer patients before treatment are higher than those of healthy people. Moreover, sCD163 levels may be related to tumor load. After the treatment, sCD163 levels may decrease. The downward trend in sCD163 levels may vary from tumor to tumor and from treatment to treatment, but these conclusions need to be confirmed by more studies.

Four of the eight articles included determined that the baseline level of sCD163 was associated with tumor stage. Higher levels of sCD163 were associated with higher tumor stage [multiple myeloma (12), gastric cancer (15), hepatocellular carcinoma (9) and epithelial ovarian cancer (10)]. The other two studies demonstrated no association with the stages [hepatocellular carcinoma (17) and classical Hodgkin lymphoma (14)]. Since most studies only mentioned the relationship between baseline levels and disease outcomes before treatment, more studies are needed to confirm the value of sCD163 as a target for disease monitoring in subsequent disease progression. Konstantin Kazankov’s study showed sCD163 levels throughout the follow-up period (increased in the first week after treatment, decreased after 4 weeks, and increased slightly after 12 weeks). The changes of sCD163 did not exhibit a reliance either on the treatment type or on the stage of baseline BCLC (9).

The limitations of this meta-analysis warrant mentioning. First, only eight studies including 1,236 patients participated in this meta-analysis and thus the research p applied in the subgroup analyses can be considered insufficient this prevents comprehensive verification of the observed tumor relationship. Second, the cut-off values were not available in two of the studies and the definition of cut-off values for the serum sCD163 in different research is non-uniform. In addition, the demographics of more than a half of the studies selected have a highly skewed gender. Unfortunately, adjusted estimates could not be performed in this meta-analysis without enough data for the adjustment by gender. Consequently, more multicentered, homogenous, and larger sample studies are needed to produce more accurate conclusions.

## Conclusion

The level of serum sCD163 in multiple cancers predicted poor OS and poor PFS. Serum sCD163 might be a novel prognostic biomarker in cancers. More high-quality studies with a larger sample size and a standardized methodological design are required to further certify the prognostic value of serum sCD163 in cancers.

## Data Availability Statement

The original contributions presented in the study are included in the article/**Supplementary Material**. Further inquiries can be directed to the corresponding author.

## Author Contributions

SQ, HZ, and HD initiated this study and participated in its design. SQ, HZ, and BM performed study selection, data extraction, and data analysis. QS and HZ drafted the manuscript. FT and XuS supervised all aspects of the study. All authors contributed to the article and approved the submitted version.

## Funding

This work was supported by the National Science Foundation of China (81573770).

## Conflict of Interest

The authors declare that the research was conducted in the absence of any commercial or financial relationships that could be construed as a potential conflict of interest.
