# Supplementary material for: Is sCD163 a Clinical Significant Prognostic Value in Cancers? A Systematic Review and Meta-Analysis
Source: Front Oncol. 2020 Nov 10;10:585297. doi: 10.3389/fonc.2020.585297 (PMC7683770; doi:10.3389/fonc.2020.585297)
Supplement: Supplementary file 1 [file Table_1.docx]

***Supplementary Materials***

**Table 1.** Search strategies

| Databases | Search items | Result |
| --- | --- | --- |
| PubMed | #1 (((("Neoplasms"[Title/Abstract] OR "Neoplasia"[Title/Abstract]) OR "Tumor"[Title/Abstract]) OR "Cancer"[Title/Abstract]) OR "Malignancy"[Title/Abstract]) OR (((("neoplasm s"[All Fields] OR "Neoplasms"[MeSH Terms]) OR "Neoplasms"[All Fields]) OR "neoplasm"[All Fields]) AND "Malignancy"[Title/Abstract])  #2 "CD163"[Title/Abstract] OR "sCD163"[Title/Abstract] OR "soluble cd163"[Title/Abstract] OR "soluble scavenger receptor"[Title/Abstract]  #3 (#1) AND (#2) | 1209 |
| the Web of Science | TS= (Neoplasms OR  Neoplasia  OR  Tumor  OR  Cancer  OR  Malignancy  OR  Neoplasms  Malignancy)  AND  TS= (CD163 OR  sCD163  OR  soluble  CD163  OR  soluble  scavenger  receptor) | 1380 |
| Embase | #1 (Neoplasms OR  Neoplasia  OR  Tumor  OR  Cancer  OR  Malignancy  OR  Neoplasms  Malignancy):ab ti  #2 (CD163 OR  sCD163  OR  soluble  CD163  OR  soluble  scavenger  receptor):ab ti  #3 #1 AND #2 | 7 |
| CBM | ("癌"[常用字段:智能] OR "肿瘤"[常用字段:智能]) AND ( "CD163"[常用字段:智能] OR "可溶性清道夫受体"[常用字段:智能]) OR "sCD163"[常用字段:智能] | 332 |
| Scopus | [TITLE-ABS-KEY (neoplasms OR neoplasia OR tumor OR cancer OR malignancy OR neoplasms AND malignancy) AND TITLE-ABS-KEY (cd163 OR scdgf OR soluble AND cd163 OR soluble AND scavenger AND receptor )](https://www.scopus.com/results/documentSpellSuggest.uri?sort=plf-f&src=s&sid=219ec72cd1444a682934038baef3169f&sot=a&sdt=a&sl=181&s=TITLE-ABS-KEY+%28+Neoplasms+OR+Neoplasia+OR+Tumor+OR+Cancer+OR+Malignancy+OR+Neoplasms+Malignancy+%29+AND+TITLE-ABS-KEY+%28+CD163+OR+scdgf+OR+soluble+CD163+OR+soluble+scavenger+receptor+%29&origin=resultslist) | 13 |
| Cochrane | Neoplasms OR Neoplasia OR Tumor OR Cancer OR Malignancy OR Neoplasms Malignancy in Title Abstract Keyword AND CD163 OR sCD163 OR soluble CD163 OR soluble scavenger receptor in Title Abstract Keyword | 67 |

**
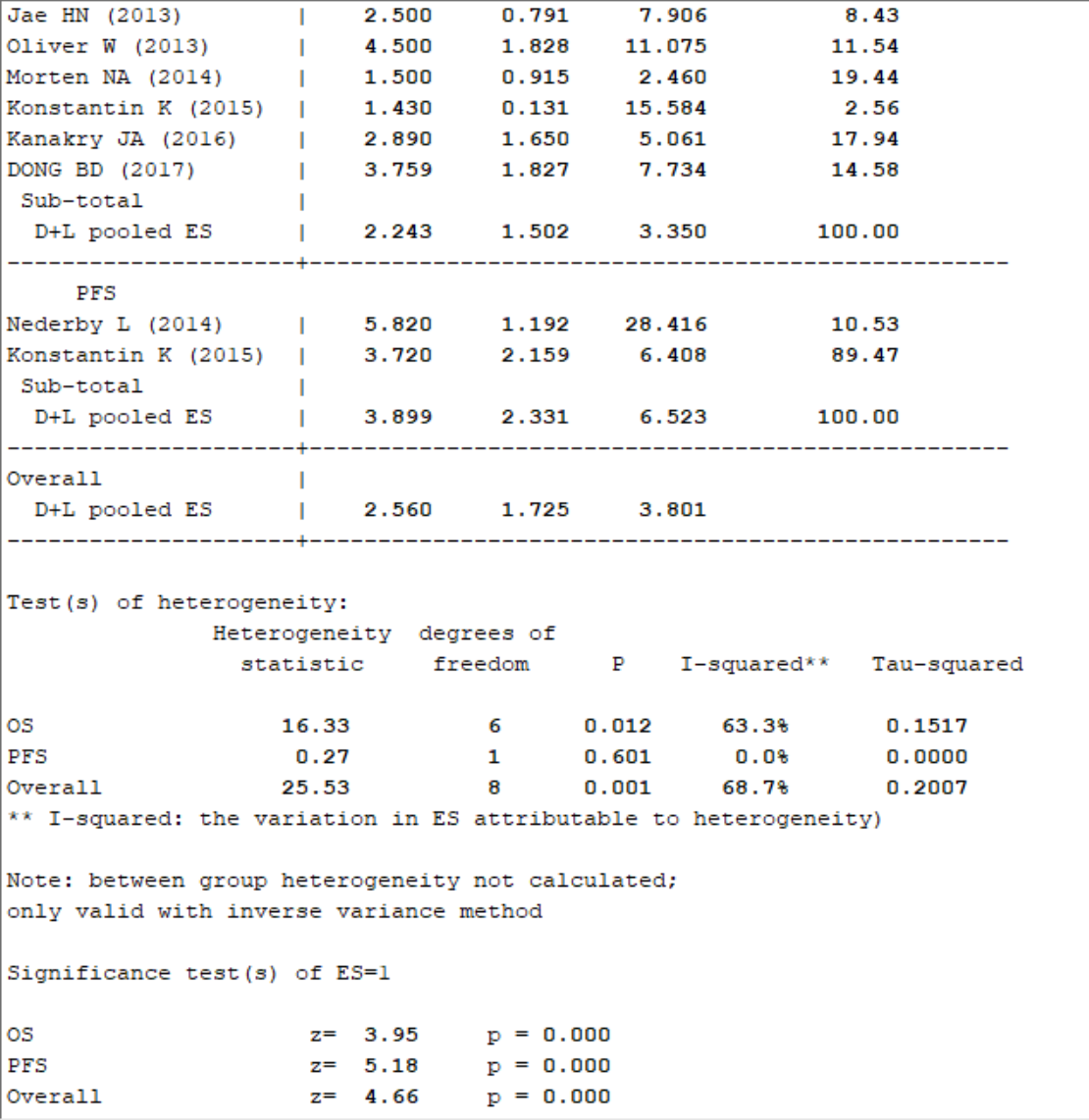
**

**Supplementary Figure 1.** Meta-analysis result of subgroup analysis of survival outcome


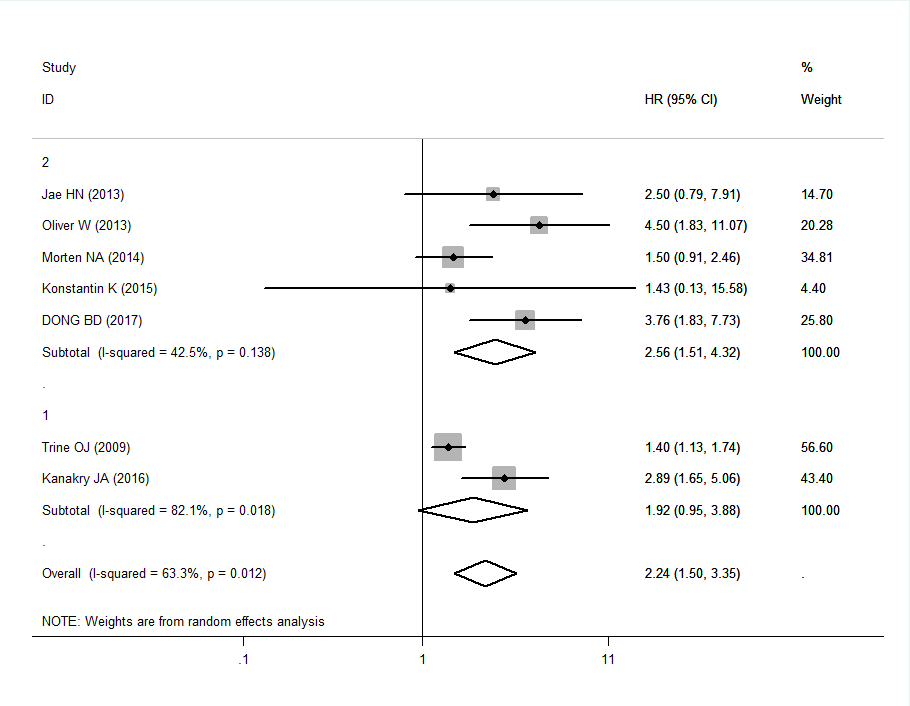


**Supplementary Figure 2.** Meta-analysis result of subgroup analysis of sample size using random-effects model


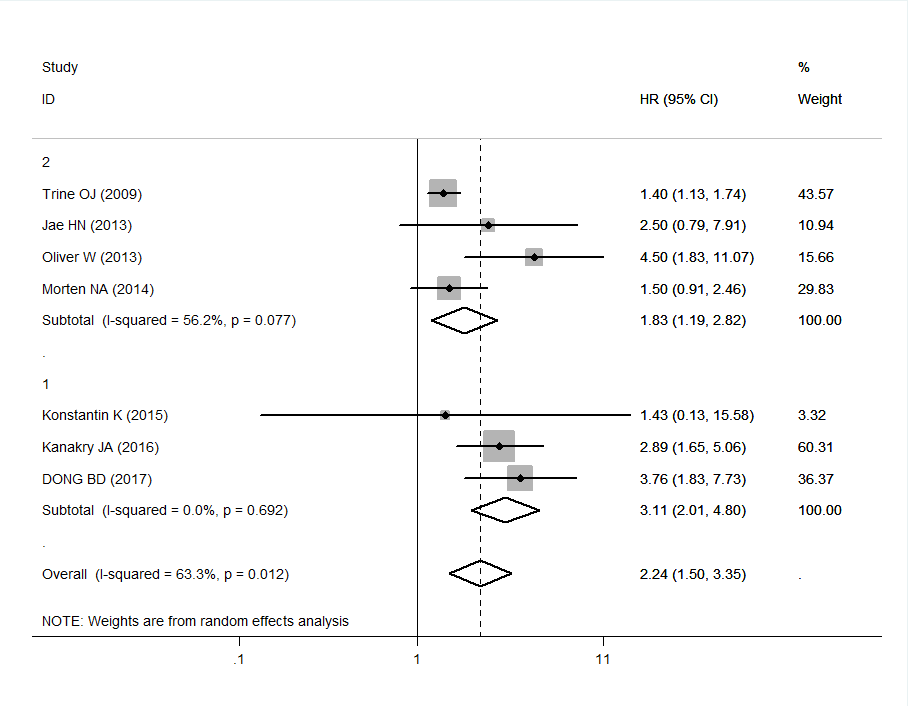


**Supplementary Figure 3.** Meta-analysis result of subgroup analysis of analysis method using random-effects model


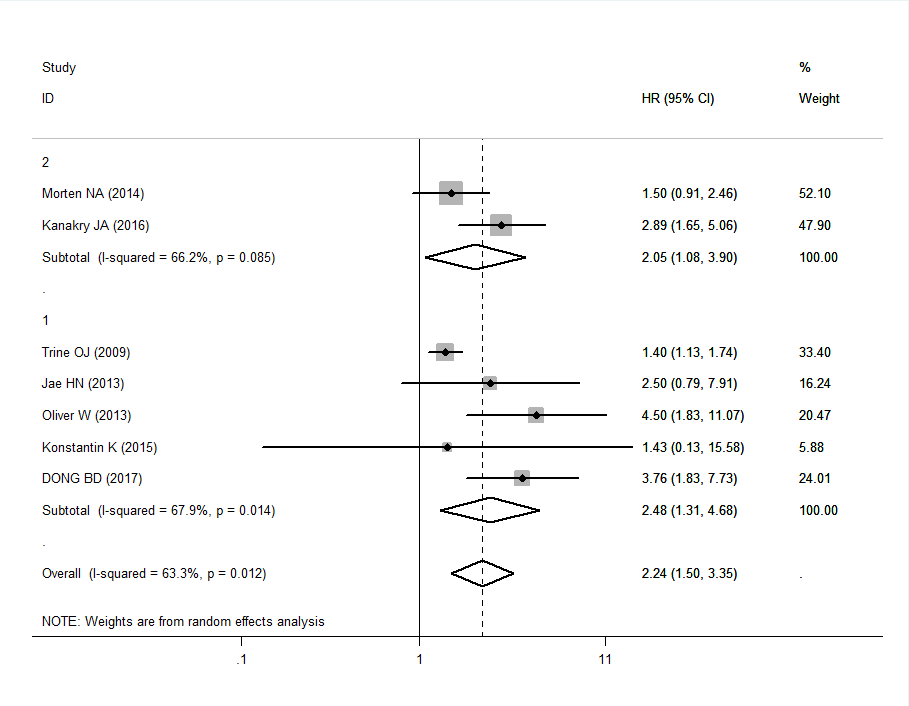


**Supplementary Figure 4.** Meta-analysis result of subgroup analysis of cancer type using random-effects model


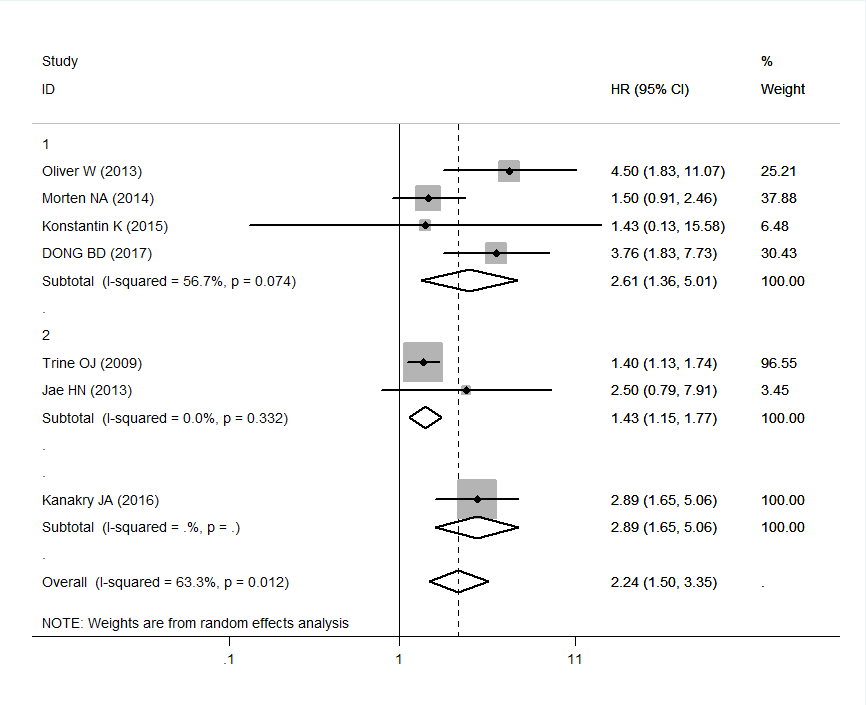


**Supplementary Figure 5.** Meta-analysis result of subgroup analysis of age at diagnosis using random-effects model

**
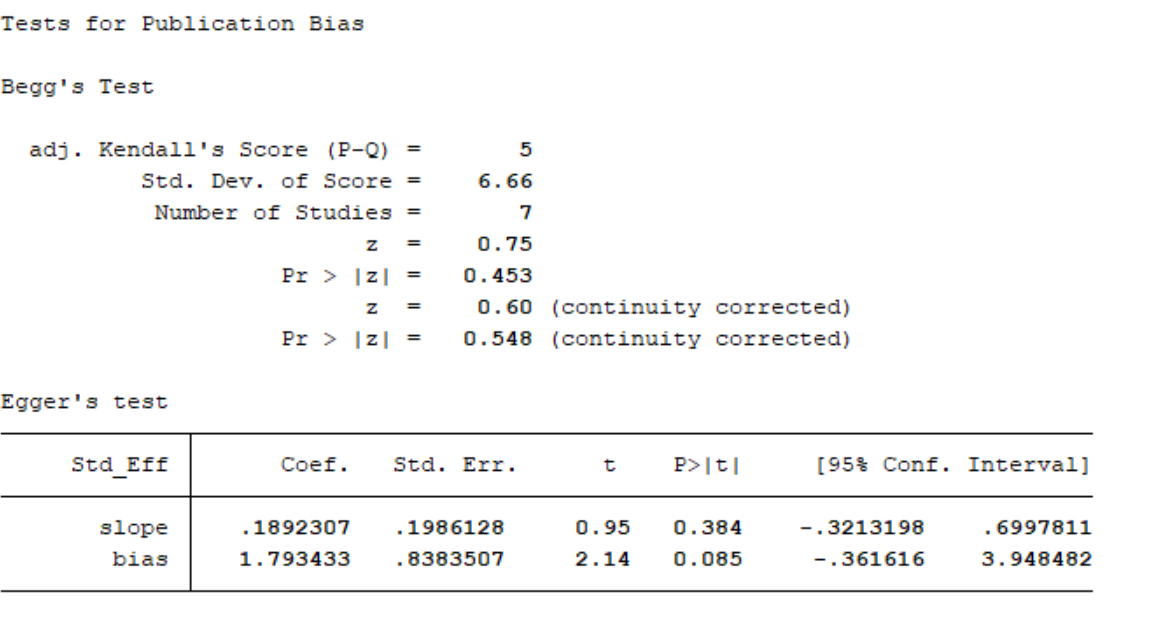
**

**Supplementary Figure 6.** Begg’s test for publication bias of results of survival


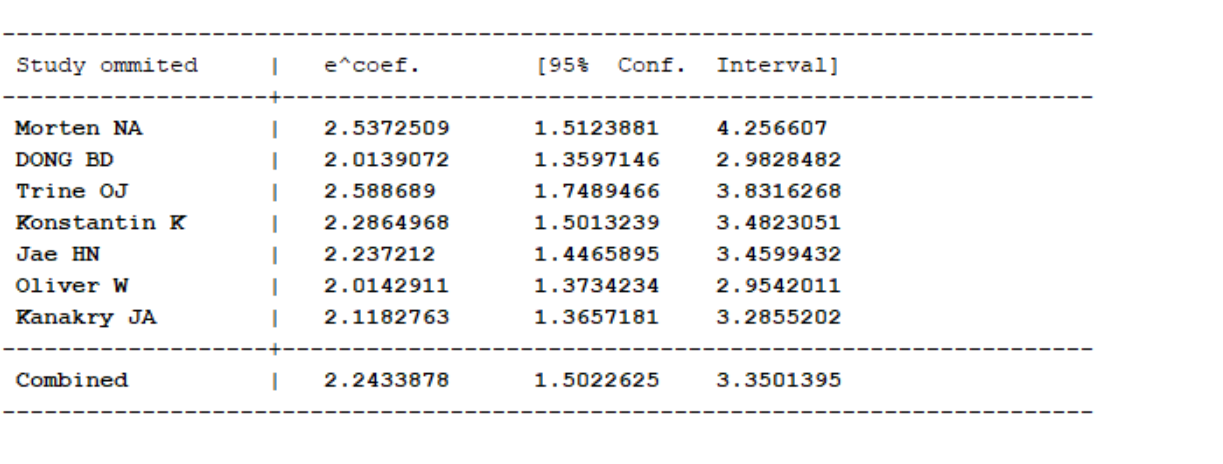


**Supplementary Figure 7.** Sensitivity analysis for studies about OS by omitting each study sequential


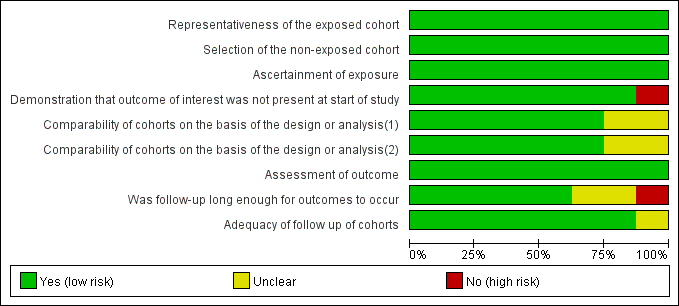


**Supplementary Figure 8.** Assessment analysis for studies by the Newcastle-Ottawa Scale (NOS)
